# The evolution of iPSC-based cell therapy across autologous and allogeneic pathways

**DOI:** 10.1016/j.xcrm.2026.102992

**Published:** 2026-08-18

**Authors:** Anmin Wang, Yunpei Zhang, Hongkui Deng, Mingxia Gu

**Affiliations:** 1 Department of Anesthesiology and Perioperative Medicine, David Geffen School of Medicine, Eli and Edythe Broad Center of Regenerative Medicine and Stem Cell Biology, University of California, Los Angeles, CA 90095, USA; 2 School of Basic Medical Sciences, MOE Engineering Research Center of Regenerative Medicine, State Key Laboratory of Natural and Biomimetic Drugs, Peking University Health Science Center and the MOE Key Laboratory of Cell Proliferation and Differentiation, College of Life Sciences, Peking-Tsinghua Center for Life Sciences, Peking University, Beijing 100191, China; 3 Changping Laboratory, Beijing 102206, China

**Keywords:** induced pluripotent stem cells, iPSCs, iPSC-based cell therapy, autologous transplantation, allogeneic transplantation, reprogramming, hypoimmune engineering, HIP engineering, regenerative medicine, clinical translation

## Abstract

Induced pluripotent stem cell (iPSC)-based treatments have revolutionized regenerative medicine, yielding patient-specific renewable cells without raising ethical concerns related to the use of embryonic stem cells. In two decades, iPSC therapies have advanced from basic research to clinical trials and official approvals. Early research focused on autologous transplantation, exemplified by the 2014 retinal pigment epithelium (RPE) graft. Yet, personalized production obstacles prompted allogeneic schemes relying on HLA cell banks and immunomodified donor cells. Meanwhile, innovations such as chemical reprogramming have revitalized autologous strategies. Today, autologous and allogeneic iPSC therapies complement each other for distinct clinical demands. This review traces key field milestones and analyzes prospects and hurdles for next-generation iPSC therapies.

## Graphical abstract

**Figure:**
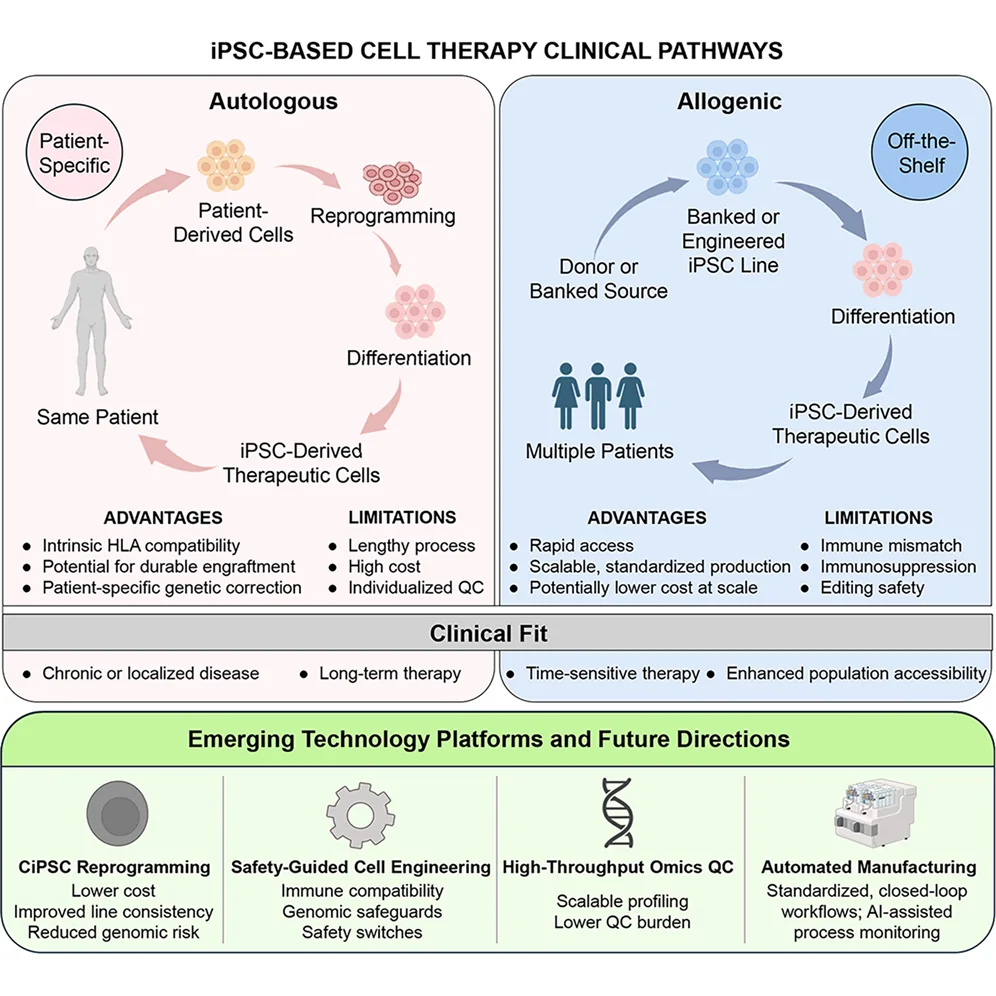

## Teaser

Wang et al. review the evolution of iPSC-based cell therapy, from early proof-of-concept studies to clinical translation and regulatory approval, showing how advances in reprogramming, manufacturing, and immune engineering have shaped autologous and allogeneic iPSC therapies into complementary clinical platforms.

## Introduction

Induced pluripotent stem cells (iPSCs) are somatic cells that have been reprogrammed back to a pluripotent state, thereby acquiring the capacity for unlimited self-renewal and the potential to differentiate into derivatives of all three germ layers.^1^^,^^2^^,^^3^^,^^4^^,^^5^ This concept was first demonstrated by Takahashi and Yamanaka in 2006, who showed that ectopic expression of four transcription factors, Oct3/4, Sox2, Klf4, and c-Myc, collectively known as OSKM, could convert mouse embryonic and adult fibroblasts into embryonic stem cell (ESC)-like cells, which they termed iPSCs.^1^ In 2007 and 2008, this breakthrough was rapidly extended to human cells by three independent groups: Yamanaka, Thomson, and Daley.^6^^,^^7^^,^^8^ These studies generated human iPSC lines that closely resembled human ESCs in morphology, gene expression profile, and trilineage differentiation potential. By decoupling pluripotency from embryo-derived materials, iPSCs created a route to patient-specific cell sources while bypassing many of the ethical concerns that had constrained ESC research. These features quickly established iPSCs as a foundational platform for human disease modeling, drug discovery, and toxicity testing, enabling patient-specific models of diverse disorders such as neurodegenerative disease, cardiomyopathy, and hematologic malignancy.^9^^,^^10^

Beyond their applications in disease modeling and drug discovery, iPSCs opened a new avenue for regenerative medicine by providing a renewable source of cells for tissue repair and replacement. Because iPSCs can be differentiated into virtually any somatic lineage, they offer a versatile platform for generating therapeutic cell products. Early enthusiasm centered on autologous transplantation, in which patient-derived iPSCs could generate genetically matched grafts for cell replacement and tissue reconstruction. This approach is particularly attractive for chronic, progressive diseases in which durable engraftment is desirable but prolonged systemic immunosuppression is clinically burdensome. In principle, autologous iPSC-derived grafts may provide HLA compatibility and long-term persistence while avoiding many of the ethical concerns associated with embryo-derived cells.^11^^,^^12^ As the field matured, advances in cell banking, manufacturing, differentiation, and gene engineering established allogeneic iPSC therapies as a scalable alternative capable of supporting broader clinical deployment. Together, these developments positioned iPSCs as a unique platform from which both autologous and allogeneic therapeutic strategies could emerge.

As the field progressed from proof-of-concept studies toward clinical translation, a series of scientific, manufacturing, and regulatory challenges came into focus. Early reprogramming methods relied on integrating viral vectors and oncogenic factors, raising concerns about insertional mutagenesis and tumorigenicity.^13^ At the differentiation stage, early protocols often generated heterogeneous products containing immature cells and residual undifferentiated iPSCs, raising the risk of teratoma formation.^14^^,^^15^^,^^16^ Beyond these biological challenges, autologous manufacturing introduced substantial operational complexity, as each patient-specific line required individualized production, extensive quality control testing, prolonged timelines, and considerable cost.^17^^,^^18^ These limitations temporarily shifted attention toward more “off-the-shelf” allogeneic strategies, including HLA-haplobanked iPSC lines and gene-edited universal donor platforms.^19^^,^^20^ At the same time, they stimulated advances in reprogramming, differentiation, manufacturing, and quality control technologies that continued to strengthen the broader cell therapy field.

The resulting advances reshaped both translational pathways. More recently, chemical reprogramming and improved differentiation technologies have renewed interest in autologous iPSC therapies, particularly for indications in which durable engraftment and immune compatibility are major therapeutic considerations.^21^^,^^22^^,^^23^ At the same time, advances in cell banking, immune engineering, and large-scale manufacturing have accelerated the development of allogeneic iPSC therapies, enabling scalable off-the-shelf products and the first regulatory approvals.^24^ Autologous and allogeneic approaches now represent two translational routes within iPSC-based therapy, and each of these offers distinct advantages and limitations depending on the clinical context. In this review, we trace the evolution of both pathways, from early autologous transplantation and the emergence of allogeneic cell banking to recent advances in immune engineering, chemical reprogramming, and clinical translation, examining how each milestone has shaped the current landscape of iPSC-based therapy (Figure 1).

**Figure 1:**
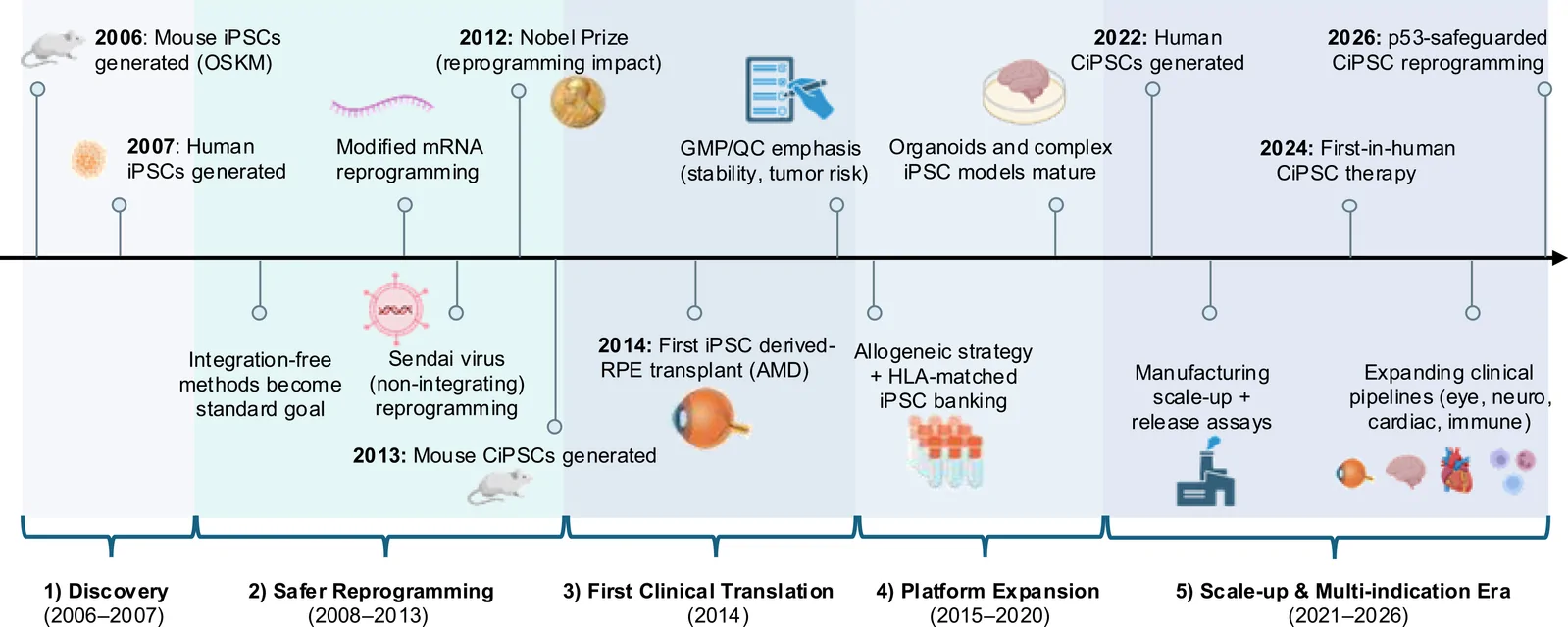
Milestones in iPSC reprogramming and clinical translation (2006–2026) The timeline depicts five stages in the evolution of iPSC-based cell therapy: discovery, safer reprogramming, initial clinical translation, platform expansion, and the emergence of scalable, multi-indication therapeutic pipelines. Major milestones include the generation of mouse and human iPSCs; the development of integration-free reprogramming methods; recognition of cellular reprogramming by the 2012 Nobel Prize; the generation of mouse and human chemically induced pluripotent stem cells (CiPSCs); increasing emphasis on good manufacturing practice and quality control (GMP/QC); the first autologous iPSC-derived retinal pigment epithelium (RPE) transplantation in 2014; HLA-matched iPSC banking; advances in organoid differentiation and manufacturing scale-up; and the expansion of clinical programs across ophthalmic, neurological, cardiac, endocrine, and immune-cell applications. AMD, age-related macular degeneration; T1DM, type 1 diabetes mellitus.

## Early iPSC translation: Autologous therapy as the initial clinical paradigm (2006–2014)

The first decade of iPSC translation was driven less by a choice between autologous and allogeneic approaches than by a more fundamental challenge: establishing whether iPSC-derived products could be developed into safe and effective clinical therapies at all. First, reprogramming methods needed to preserve genomic integrity while minimizing oncogenic risk. Second, differentiation protocols had to generate sufficiently pure, mature, and lineage-specific cell products suitable for transplantation. Third, the immune behavior of iPSC-derived grafts had to be clarified, including whether the reprogramming process itself might introduce novel immune targets.^25^

### Reprogramming: Reducing genomic and oncogenic risks

Reprogramming safety represented the first major barrier to clinical translation, as early iPSC derivation relied on integrating viral vectors that introduced risks of insertional mutagenesis, transgene reactivation, and tumorigenicity.^26^^,^^27^^,^^28^^,^^29^ These risks drove the development of transient, non-integrating approaches, among which episomal plasmid DNA, Sendai virus, and synthetic modified mRNA became the dominant platforms for clinical-grade iPSC derivation.^30^^,^^31^^,^^32^^,^^33^^,^^34^^,^^35^ Compatible with good manufacturing practice (GMP) workflows and scalable cell line production, these methods established a safer technical foundation for therapeutic development.^36^^,^^37^ The transition away from integrating vectors was, therefore, not simply a methodological improvement but a prerequisite for clinical translation.

### Differentiation: Achieving lineage identity, maturity, and product control

Once safer reprogramming became feasible, the next challenge was to determine whether iPSCs could be differentiated reproducibly into therapeutic cell lineages. This required more than the generation of lineage-marker-positive cells *in vitro*. A clinically useful product had to be appropriately specified, sufficiently mature and functional, and depleted of residual undifferentiated cells that could form teratomas after transplantation.

Among candidate iPSC-derived lineages, retinal pigment epithelium (RPE) had several biological and operational features that made it especially suitable for early clinical translation. Established differentiation protocols could generate enriched RPE monolayers with characteristic pigmentation, cobblestone morphology, polarized secretion, and expression of mature RPE markers, providing accessible criteria for product characterization and release.^38^ As a relatively simple monolayer epithelium, RPE also avoided the need for complex three-dimensional tissue reconstruction or long-range integration into host circuitry.^17^^,^^18^^,^^39^^,^^40^^,^^41^ Together, these features made RPE the first lineage to meet the requirements for clinical translation and a tractable initial target for iPSC-derived therapy.

By contrast, dopaminergic neurons for Parkinson’s disease illustrate why some iPSC-derived lineages required a longer path to clinical translation. Unlike RPE, these cells must acquire a precise regional identity and then functionally integrate into host neural circuits after transplantation. Developmentally guided protocols, therefore, became important because they improved the reproducible generation of midbrain dopaminergic progenitors, replacing earlier, less specific neural differentiation methods.^42^ Yet, even with improved differentiation, these products had to meet additional functional requirements after transplantation, including survival, maturation, axonal outgrowth, circuit integration, and functional recovery without overgrowth or inappropriate differentiation. Together, early differentiation studies have established a central lesson for iPSC therapy: clinical-grade production was achievable, but each therapeutic cell type required its own standards for identity, purity, maturity, potency, and safety.

### Immunogenicity: Testing the autologous premise

In parallel with advances in reprogramming and differentiation, the field also had to test a central assumption of autologous therapy: whether patient-derived iPSC grafts would behave immunologically as self-tissue. Early studies questioned this premise by suggesting that syngeneic iPSC derivatives could provoke immune responses, potentially through aberrant gene expression or mutation-derived immune targets.^25^^,^^43^ These findings raised concern that autologous iPSC products might not automatically evade immune recognition. Subsequent studies, however, showed that intrinsic immunogenicity was not a general property of properly differentiated iPSC derivatives.^44^^,^^45^ Syngeneic iPSC-derived skin, bone marrow, and other tissues engrafted in mice without greater rejection than comparable ESC-derived tissues, indicating that the differentiation state and product quality were the key determinants of immune behavior.^44^^,^^45^

Non-human primate studies further strengthened the rationale for autologous therapy, especially in the central nervous system.^46^^,^^47^^,^^48^^,^^49^ In rhesus monkeys lesioned with 1-methyl-4-phenyl-1,2,3,6-tetrahydropyridine (MPTP), a neurotoxin used to model parkinsonian dopaminergic injury, autologous iPSC-derived neural progenitors survived after transplantation, with only minimal inflammatory responses.^47^ Later studies in monkeys showed that autologous iPSC-derived dopaminergic neurons supported long-term innervation and behavioral improvement without immunosuppression, graft overgrowth, or tumor formation.^46^^,^^47^^,^^49^ A direct comparison further showed that non-immunosuppressed autologous graft recipients had more sustained functional improvement, stronger graft survival, and more extensive axonal outgrowth than non-autologous recipients on immunosuppression.^48^^,^^50^ These findings supported the immunological rationale for autologous iPSC therapy in the central nervous system, but their generalizability was unclear. Much of the strongest evidence came from grafts placed in the striatum, a partially immune-privileged compartment. Whether comparable immune tolerance can be achieved in tissues with stronger immune surveillance, including endocrine tissues, remains less certain.^51^

### Autologous iPSC-RPE: The first clinical test of iPSC translation

Advances in reprogramming safety, differentiation control, and immune characterization had made autologous iPSC-derived RPE transplantation feasible as a first clinical application.^39^ Because wet age-related macular degeneration represented a largely unmet clinical need and iPSC-derived RPE had been shown to recapitulate key functional properties of native RPE, it provided a fitting clinical context for this first test of iPSC-derived RPE transplantation.^39^ In September 2014, Mandai and colleagues transplanted an autologous iPSC-derived RPE sheet measuring 1.3 × 3.0 mm into a patient with neovascular (wet) AMD, marking the first clinical use of an iPSC-derived product worldwide.^17^ Early and long-term follow-up supported the safety of this first-in-human application, showing stable visual function, graft survival, and no safety-related adverse events or need for systemic immunosuppression.^17^^,^^18^ This study demonstrated that an autologous iPSC product could be generated, quality controlled, transplanted, and maintained safely in a human recipient. Together, these findings established early clinical proof-of-concept for the autologous iPSC therapeutic paradigm.

## Reorientation: Operational bottlenecks and the emergence of allogeneic translation (2015–2019)

The first autologous iPSC-RPE transplantation demonstrated that iPSC-derived products could be manufactured and transplanted safely in humans. However, the preparation of a second patient-specific product revealed a different challenge: operational scalability. Each autologous iPSC line carried its own manufacturing, genomic, regulatory, and release-decision burden, making clinical translation far more complex than the success of a single transplantation might suggest.^52^ As a result, the central question facing the field after 2015 was no longer simply whether an autologous iPSC-derived product could be generated and transplanted safely, but whether such therapies could be produced reliably, affordably, and at a scale suitable for broader clinical use.

### The 2015 suspension: Genomic uncertainty and the burden of individualized manufacturing

In 2015, during preparation of an autologous iPSC-RPE product for a second patient, whole-genome sequencing identified copy number alterations in the patient-derived iPSCs and differentiated RPE cells that were not detected in the original somatic cells, including alterations affecting a known oncogene.^52^ Although subsequent analyses suggested that the oncogenic risk associated with these alterations was likely low, no validated framework existed at the time for interpreting such genomic findings and translating them into a clinical release decision for a patient-specific iPSC product.^17^ The team, therefore, elected not to proceed with the second transplantation, and the program was suspended.

The significance of this episode was not that autologous iPSC-RPE therapy had failed biologically. Rather, it exposed a new set of challenges facing individualized manufacturing. Under the autologous approach, each patient required a complete production cycle, including tissue biopsy, reprogramming, iPSC line establishment, expansion, differentiation, characterization, and release testing. At the time, this process lasted approximately one year and cost about 100 million yen (roughly US$1 million) per patient.^19^^,^^53^ More importantly, each cycle required independent quality control, including whole-genome sequencing, without the ability to carry forward characterization data from one patient to the next.^54^

In this sense, every autologous cell product required the characterization and qualification of a newly generated iPSC line. Copy number alterations, point mutations, and other acquired abnormalities may arise during reprogramming and expansion, and their clinical relevance can vary by genomic context, affected locus, cell type, and intended use. For a patient-specific product, each finding must, therefore, be interpreted in real time, with long-term assessments before clinical release being difficult. While complete patient specificity is the defining advantage of autologous therapy, this advantage would become achievable only by addressing operational challenges of successful and consistent donor-by-donor manufacturing, testing, interpretation, and release. The 2015 suspension, therefore, marked a turning point, highlighting genomic quality control as a central challenge for scalable autologous iPSC manufacturing, rather than simply a final release criterion.

### HLA-haplobanking: Converting individualized manufacture into shared infrastructure

The release of the first clinical-grade iPSC lines from the CiRA iPSC Stock Project at Kyoto University in August 2015 established an alternative model for overcoming the constraints of individualized manufacturing.^19^ Instead of generating a new iPSC line for each patient, the CiRA strategy used HLA-homozygous donors to create banked iPSC lines that could be matched to multiple recipients. These donors were recruited through the Japanese Red Cross Society, the Japan Marrow Donor Program, and public cord blood banks. Because HLA-homozygous donors carry the same major HLA haplotype on both chromosomes, their derived iPSC lines express a single major HLA haplotype, simplifying donor-recipient matching.^19^

This design enabled partial immune matching at population scale. A heterozygous recipient who carries one of the donor haplotypes can be matched to donor-derived cells at the major HLA loci, even though the recipient’s second haplotype is not represented in the graft. By prioritizing the most common haplotypes in the target population, a relatively small number of donor lines can, therefore, cover a substantial fraction of recipients. The first three released CiRA lines collectively matched approximately 32% of the Japanese population, and by 2022, the haplobank had expanded to cover approximately 40%.^55^^,^^56^

The clinical effect of this model was immediate. The first iPSC-related allogeneic transplantation, again involving an RPE product, used HLA-matched cells from the CiRA stock.^19^^,^^57^ Compared with the autologous RPE workflow, the preparation time fell from approximately 1 year to roughly 1 month, while per-patient cost declined from nearly 100 million yen to under 20 million yen, or roughly US$200,000.^19^ HLA-haplobanking, therefore, replaced patient-by-patient manufacturing with a shared cell source infrastructure, offering substantial logistical and economic advantages.^19^^,^^57^

The advantages of this model extend beyond a single clinical program. A master cell bank can be extensively characterized once, including whole-genome sequencing, single-cell transcriptomic profiling, tumorigenicity testing, and other release assays, with these qualification data subsequently supporting treatment of multiple recipients.^58^ This shared characterization framework reduces the need for repeated patient-specific qualification and creates opportunities for more standardized and scalable quality control strategies. Importantly, this genomic characterization is increasingly supported by computational and bioinformatic tools, such as copy number inference, recurrent-mutation detection, and standardized genomic stability scoring, that screen iPSC lines and their derivatives for acquired abnormalities more rapidly and at a lower cost than whole-genome sequencing alone.^54^^,^^58^ Manufacturing can also be organized around standardized batches, rather than one-off patient-specific products, reducing the per-patient production burden and making off-the-shelf supply more feasible. For indications that require rapid treatment, consistent dosing, or broad distribution, this model provides advantages that autologous manufacturing cannot easily match.^19^^,^^57^

These advantages, however, also come with important caveats. The efficiency of HLA matching depends strongly on population genetics. A haplobank can cover a relatively large fraction of recipients in a genetically homogeneous population, but comparable coverage in more diverse or admixed populations would require many more donor lines.^59^^,^^60^ Crucially, HLA matching also does not completely eliminate immune risks. Even when major HLA loci are matched, differences at minor histocompatibility loci and other non-HLA polymorphisms may still trigger low-grade or chronic rejection.^60^^,^^61^ As a result, most clinical allogeneic iPSC programs continue to include at least short-course immunosuppression, with duration varying by indication. Donor recruitment, consent, and long-term cell line use are also governed by the ethical and regulatory frameworks that vary across jurisdictions, making international standardization difficult.^58^

By the end of this period, HLA-haplobanking had established allogeneic translation as a practical route toward scalable iPSC-based therapy. While the biological appeal of autologous products remained intact, the field’s focus expanded beyond proof-of-concept to questions of manufacturing, qualification, and clinical deployment at scale. Shared donor lines, therefore, emerged as a key foundation for subsequent iPSC-based clinical development.^19^^,^^57^^,^^59^

## Maturation of two translational pathways: Allogeneic acceleration and autologous revival (2019–present)

The years following the 2015 pivot were shaped by two major technological advances: increasingly mature differentiation protocols and programmable genome editing. Improved differentiation protocols expanded the range of therapeutic cell types that could be produced with clinical-grade identity, purity, and potency, thereby supporting both autologous and allogeneic iPSC-based therapies.^20^^,^^62^^,^^63^^,^^64^ At the same time, programmable genome editing enabled precise modification of iPSC lines at HLA loci and immune-regulatory pathways. While this capability could be applied to both approaches, its translational impact was particularly pronounced in allogeneic development, where the costs of cell line engineering, process qualification, and release testing could be distributed across many recipients through a shared master cell bank.

### Allogeneic acceleration: Genome editing and differentiation maturation

The contribution of programmable genome editing, particularly CRISPR-Cas9, was to move allogeneic translation beyond donor selection toward active immune engineering of iPSC lines. Instead of relying solely on HLA matching, edited cells could be designed to remove major immune targets or express inhibitory signals that protect the graft from immune attack. This strategy gave rise to hypoimmune (HIP) engineering, which aims to generate broadly compatible donor cells for allogeneic therapy.^20^^,^^62^ In 2019, Deuse and colleagues reported that human and mouse iPSC lines engineered with biallelic knockout of B2M and CIITA, together with overexpression of CD47, could evade both adaptive and innate immune surveillance in fully immunocompetent allogeneic recipients.^20^ In this design, B2M deletion reduces CD8^+^ T cell allorecognition by removing HLA class I expression, CIITA deletion limits CD4^+^ T cell recognition by suppressing HLA class II expression, and CD47 overexpression helps counteract innate immune clearance of HLA-deficient cells. Subsequent studies extended HIP editing to multiple iPSC-derived lineages and demonstrated long-term graft survival in non-human primates.^62^ Clinical proof of principle came from Sana Biotechnology’s UP421 program.^65^ In the initial report of this first-in-human study, HIP-edited primary allogeneic islet cells survived and produced detectable glucose-responsive C-peptide for 12 weeks without systemic immunosuppression. Although a marginal mass of partially HIP-edited primary islets rather than a therapeutic dose of HIP iPSC-derived islet cells was transplanted in this study, it provided an important signal for universal iPSC-derived HIP therapeutic programs.

### Differentiation maturation: Expanding the therapeutic landscape

The maturation of differentiation protocols represented a second major advance, enabling the reproducible conversion of iPSCs into therapeutic cell products with increasingly well-defined identity, purity, and potency. As protocols became more developmentally informed, investigators gained greater control over lineage specification, cellular maturity, functional competence, and depletion of off-target populations. Improved small-molecule screening, lineage-marker-based purification, and single-cell transcriptomic profiling further standardized intermediate and final cell states, moving the field from laboratory-specific protocols toward reproducible clinical-grade manufacturing. Computational classifiers benchmarked against reference cell atlases now help identify optimal differentiation states for transplantation.^66^ As a result, clinically tractable products have emerged across multiple lineages, including dopaminergic progenitors for Parkinson’s disease, islet-like endocrine structures for diabetes, cardiomyocyte spheroids and sheets for heart failure, and iPSC-derived natural killer and engineered T cells for oncology.^63^^,^^64^^,^^67^^,^^68^^,^^69^ These advances benefited both autologous and allogeneic development. For autologous approaches, improved differentiation enhanced the reliability, consistency, and functional performance of patient-specific products. For allogeneic platforms, standardized differentiation protocols enabled scalable manufacturing from shared donor lines and master cell banks. Together, these developments established differentiation as a central foundation for clinical translation across multiple therapeutic areas.

The clinical impact of these advances became increasingly evident during this period. In Parkinson’s disease, a phase 1/2 trial transplanted allogeneic iPSC-derived dopaminergic progenitors into seven patients. Among the six patients evaluable for efficacy, four showed motor improvement. The study also reported no tumor formation, together with evidence of graft survival and dopamine production.^70^ These data supported the conditional and time-limited approval of AMCHEPRY/raguneprocel by Japan’s Ministry of Health, Labour and Welfare on March 6, 2026.^24^ In the same approval cycle, RiHEART/IPSOC-1, Cuorips Inc.’s allogeneic iPSC-derived cardiomyocyte sheet product for severe heart failure, received conditional and time-limited approval.^24^ Additional programs extended iPSC-based clinical translation into diabetes and oncology, including stem-cell-derived islet approaches and iPSC-derived natural killer (NK) cell platforms.^63^^,^^65^^,^^69^

### Autologous revival: Chemical reprogramming and the reassessment of patient-specific therapy

While allogeneic programs were advancing through improvements in banking, manufacturing, and immune engineering, the reprogramming step itself was being redefined by chemical approaches. Unlike transcription factor-based approaches, chemical reprogramming uses defined small-molecule cocktails to drive somatic cells toward pluripotency without introducing exogenous genetic material. The approach was first demonstrated in mouse cells in 2013 and has since been widely adopted by different groups for small-molecule discovery, mechanistic studies, and disease modeling.^35^^,^^71^^,^^72^^,^^73^^,^^74^^,^^75^^,^^76^^,^^77^^,^^78^^,^^79^^,^^80^ Extending this strategy to human cells proved substantially more challenging, as human cells are less responsive to reprogramming cues than their murine counterparts.^81^^,^^82^^,^^83^ In 2022, Guan and colleagues reported human chemically induced pluripotent stem cells (hCiPSCs). These cells were generated from adult adipose-derived stromal cells and dermal fibroblasts by rewiring epigenetic barriers to reprogramming.^81^ Subsequent studies have expanded hCiPSC generation to additional starting materials, including peripheral blood cells and selected T cell subsets, while also reducing reprogramming timelines and broadening the range of clinically accessible cell sources.^84^^,^^85^^,^^86^ The availability of commercial reagents and standardized protocols indicates that hCiPSC generation has been achieved by several independent groups,^87^^,^^88^^,^^89^^,^^90^ although systematic cross-laboratory reproducibility has not yet been formally benchmarked. At the same time, direct comparisons with established transcription factor-based methods remain limited, and further validation is needed to determine the scalability and clinical utility of chemical reprogramming across diverse settings.

The translational importance of chemical reprogramming lies in its potential to reduce several burdens that had limited patient-specific therapy. Small-molecule cocktails are chemically defined, scalable, and compatible with clinical-grade manufacturing.^91^ They also avoid viral particle production, vector qualification, and repeated synthesis or delivery of nucleic acid reagents.^21^^,^^71^^,^^72^^,^^75^^,^^92^^,^^93^^,^^94^^,^^95^^,^^96^^,^^97^^,^^98^ A defined chemical input may also reduce run-to-run variability in delivery efficiency and reprogramming-factor stoichiometry, although direct head-to-head comparisons with conventional methods remain limited.^91^ One proposed safety-relevant distinction is that efficient chemical induction has been reported to proceed with preserved p53 activity, unlike the p53 suppression often associated with OSKM-driven reprogramming^99^; whether this represents a measurable clinical-grade genomic safety advantage, however, remains to be established in direct, GMP-grade head-to-head comparisons against episomal, Sendai virus, and mRNA reprogramming methods. This feature is relevant to the same category of genomic risk interpretation that complicated the 2015 RIKEN autologous RPE program, although its magnitude will need to be tested in systematic clinical-grade comparisons. Thus, the current question is not whether human chemical reprogramming is technically demanding, but whether its defined, programmable, and increasingly reproducible workflow can be standardized to the level required for clinical manufacturing.

hCiPSC-derived products have been reported across therapeutically relevant lineages, including pancreatic islets, hepatocytes, RPE, engineered immune cells, and neural stem cells.^63^^,^^89^^,^^90^^,^^100^^,^^101^^,^^102^^,^^103^^,^^104^^,^^105^^,^^106^^,^^107^ These studies evaluated tumorigenicity, metabolic function, transplantation performance, and donor-to-donor reproducibility in relevant experimental models. They also helped establish an alternative transplantation strategy under the abdominal rectus sheath, designed to avoid the acute graft loss associated with conventional intrahepatic islet infusion while enabling localized graft monitoring by medical imaging. On this foundation, Wang and colleagues reported the first published clinical application of an autologous hCiPSC-derived product in 2024, treating a single patient with type 1 diabetes.^107^ Autologous hCiPSC-derived islet-like cells were transplanted underneath the abdominal anterior rectus sheath. At 1 year after transplantation, the patient had achieved insulin independence from day 75 onward, maintained a time-in-target glycemic range above 98% from month 4 onward, and showed HbA1c values in the non-diabetic range.^107^ Long-term monitoring also indicated stable graft behavior. This first-in-human pilot clinical study demonstrated that a patient-specific hCiPSC-derived product could be manufactured at clinical grade, pass in-process and final release quality control tests, and confer durable metabolic benefit in a human recipient.

Although transplantation of hCiPSC-derived islet-like cells was associated with restoration of glycemic control in this patient, the immunological contribution of autologous cell therapy remains difficult to assess. The patient had previously undergone liver transplantation and was already receiving systemic immunosuppression; thus, graft survival cannot be attributed to autologous origin alone.^107^ Moreover, because type 1 diabetes has an autoimmune etiology, immunomodulation will likely be required even in an autologous context.^108^ Overall, questions of feasibility across a larger number of patients, efficacy, durability, and safety of autologous hCiPSC-derived islets will continue to be elucidated as data from larger cohorts become available in ongoing investigational new drug (IND) studies (Table 1).

**Table 1:** Summary of global clinical trials using autologous iPSC-derived therapies

Name	Product type	Method	Indication	Sponsor	Trial ID	Phase	Target enrollment	Actual dosed patients	Country	Dose	Immune suppr.	Start date	Status	Reference
PSC-RPE sheet	RPE cell sheet	E	wet AMD	RIKEN	UMIN000011929	1/2	6	1	JP	1.3 × 3 mm (no scaffold)	N	2013	C	Mandai et al.,^17^; Takagi et al.^18^; Kamao et al.,^39^; Hirami et al.^40^
DA progenitors	DA progenitors	E	PD	McLean Hospital	–	–	1	1	US	8 M	N	2017	C	Schweitzer et al.,^42^;Song et al.^111^;
iPSC-derived RPE/PLGA	RPE on PLGA scaffold	E	dry AMD	NIH/NEI	NCT04339764	1/2	20	2	US	2 × 4 mm, ∼70,000	Y	2020	A	Khristov et al.^112^
iPLAT1	platelets	E	thrombocytopenia	CiRA, Kyoto University Hospital	jRCTa050190117	1	1	1	JP	140 B in 3 doses	N	2019	C	Sugimoto et al.^113^
iPSC-RPE	RPE suspension	ND	dry/wet AMD	Beijing Tongren Hospital	NCT05445063	1	10	0	CN	ND	ND	2022	A	Zhang et al.^41^
iPSC-CL	cardiac lineage	ND	congenital heart disease	HeartWorks, Inc.	NCT05647213	1	50	ND	US	100 M	N	2023	A	Scholz et al.^114^
hCiPSC islets	islet-like cells	C	T1DM	Tianjin First Center Hospital	ChiCTR2300072200	1	3	1	CN	∼2 B cells	Y	2023	A	Wang et al.,^107^; Du et al.,^63^; Liang et al.^106^
RGB-5088	islet-like cells	C	T1DM	Hangzhou Reprogenix Bioscience	NCT06731218	1	10	ND	CN	ND	ND	2024	A	–
hiPSC-DAP	DA progenitors	ND	PD	Shanghai East Hospital	NCT06145711	1	3	ND	CN	ND	ND	2023	A	–
DA progenitors	DA progenitors	E	PD	Massachusetts General Hospital	NCT06687837	1	8	0	US	8 M and 16 M total (4 M and 8 M per side)	N	2025	A	–
ANPD001	DA progenitors	S	PD	Aspen Neuroscience	NCT06344026	1/2	9	8 (as of June 2026)	US	5 M, 10 M	N	2024	A	Emborg et al.^115^
Autologous DA neurons	DA neurons	S	PD	McLean Hospital	NCT06422208	1/2	6	2	US	ND	N	2024	A	Hallett et al.,^46^; Osborn et al.^116^
ICA-07/iPSC-DAPs	DA progenitors	ND	PD	iCamuno Biotherapeutics Ltd.	NCT06821529	1	12	ND	CN	4 M	ND	2025	X	–

Immune suppr., immune suppression; DA, dopaminergic; PD, Parkinson's disease; ND, not disclosed. Methods: E, episomal; S, Sendai virus; C, chemical reprogramming. Countries: CN, China; JP, Japan; US, United States of America. Dose is provided as number of cells, unless stated otherwise. M, million (10^6^); B, billion (10^9^). Trial status: C, completed; A, active; X, clinical trial approval obtained, trial not yet initiated; start date, trial registration date.

Taken together, chemical reprogramming has the potential to reshape the operational framework of iPSC manufacturing. In the autologous setting, where each patient requires a newly generated iPSC line, line-to-line consistency is a critical consideration. Chemical reprogramming has been reported to pass through 2C-like intermediate states with extensive epigenetic resetting,^95^^,^^109^ which could reduce line-to-line variability, although that possibility remains to be tested directly. However, these observations require validation across larger cohorts of independently derived human lines. Combined with advances in automated iPSC manufacturing,^110^ chemical reprogramming may help reduce the cost, variability, and genomic risk burden associated with individualized production. For allogeneic therapy, its non-integrative and chemically defined nature may simplify master cell bank generation and quality control, although major existing haplobanks were established using episomal methods and have not yet adopted chemical reprogramming. Whether these advantages translate into measurable improvements in cost, quality, safety, or clinical scalability remains an open question.^54^ Overall, chemical reprogramming has expanded the range of strategies available for iPSC production and renewed interest in the long-term feasibility of patient-specific cell therapies.

## The current landscape: Autologous and allogeneic iPSC therapies

Twenty years after the first iPSCs were generated, autologous and allogeneic strategies have matured into complementary clinical platforms, with major clinical programs across both routes summarized in Tables 1 and 2. The distinction that emerged after 2015 is no longer best understood as a field-wide choice between two competing routes but as an indication-specific decision shaped by manufacturing feasibility, immune-management requirements, and the strength of clinical evidence in each therapeutic area.

**Table 2:** Selected allogeneic iPSC therapeutic programs and supporting platforms highlighted in this review

Program	Cell product	Cell source/engineering	Indication	Sponsor/trial ID	Developmental stage/current status	Immunosuppression/conditioning	Primary source
AMCHEPRY (raguneprocel)	dopaminergic neural progenitor cells	allogeneic HLA-homozygous iPSC	Parkinson’s disease	Sumitomo Pharma/RACTHERA; jRCT2090220384	phase 1/2 supporting study; conditional and time-limited approval in Japan (March 6, 2026)	tacrolimus at 5–10 ng/mL, reduced by half at 12 months, and stopped at 15 months	Sawamoto et al.^70^; World’s first two iPSC therapies in Japan.^24^
RiHEART (IPSOC-1)	iPSC-derived cardiomyocyte sheets	allogeneic iPSC	severe ischemic heart failure/ischemic cardiomyopathy	Cuorips/Osaka University; NCT04696328; jRCT2052190081	phase 1 supporting study; conditional and time-limited approval in Japan (March 6, 2026)	tacrolimus + mycophenolate mofetil + prednisolone for 3 months including tapering period	Kawamura et al.^68^; World’s first two iPSC therapies in Japan.^24^
OZTx-410	iPSC-derived pancreatic islet cell sheet	allogeneic, CiRA-stock-derived iPSC	severe type 1 diabetes eligible for islet transplantation	Kyoto University Hospital/Orizuru; jRCT2053240146	phase 1/1b; recruiting/ongoing; first participant transplanted Feb 2025	systemic immunosuppression required while the graft remains functional; agents not publicly disclosed	Kyoto University Hospital announcement, 2025
HLA-iPSC-RPE	iPSC-derived RPE-cell suspension	allogeneic HLA-homozygous iPSC; HLA matched	neovascular age-related macular degeneration	RIKEN/Kobe City Eye Hospital; UMIN000026003	exploratory clinical study; completed (5 recipients); results published 2020	local steroid (intravitreous injection of triamcinolone acetonide); no systemic immunosuppression	Sugita et al.^57^
iCEPS	iPSC-derived corneal epithelial cell sheet	allogeneic, HLA-mismatched iPSC	limbal stem cell deficiency	Osaka University; UMIN000036539/jRCTa050190084	phase 1; completed (4 recipients); results published 2024	low-dose ciclosporin in participants 1–2; no systemic immunosuppression in participants 3–4	Soma et al.^117^
CiRA iPSC stock	HLA-homozygous iPSC haplobank	non-integrating, episomally reprogrammed iPSC	clinical-grade donor-cell platform	CiRA Foundation	not phase based; operational banking and distribution platform	not applicable at platform level; determined by the derived therapeutic product	Umekage et al.^19^; Yoshida et al.^55^; Hanatani et al.^56^
SC451	HIP-modified iPSC-derived pancreatic islet cells	allogeneic, O-negative HIP-modified iPSC	type 1 diabetes	Sana Biotechnology; no clinical trial ID reported	preclinical/IND-enabling; phase 1 initiation planned for 2026 but not yet reported as initiated	designed to avoid immunosuppression; has not yet been tested clinically	https://ir.sana.com/news-releases/news-release-details/sana-biotechnology-reports-first-quarter-2026-financial-results
FT819	CD19 CAR T cells	allogeneic, off-the-shelf iPSC-derived CAR T	B cell-mediated autoimmune diseases, including SLE	Fate Therapeutics; NCT06308978	phase 1; recruiting	cyclophosphamide- or bendamustine-based pre-infusion conditioning; conditioning-free cohorts included	https://clinicaltrials.ucsf.edu/trial/NCT06308978
FT522	CD19 CAR NK cells with ADR	allogeneic, off-the-shelf iPSC-derived CAR NK	relapsed/refractory B-cell lymphoma	Fate Therapeutics; NCT05950334	phase 1; completed	rituximab, with or without lymphodepleting chemotherapy	Bachanova et al.^118^
FT825/ONO-8250	HER2-targeted CAR T cells	allogeneic, off-the-shelf iPSC-derived CAR T	HER2-positive or other advanced solid tumors	Fate Therapeutics/Ono; NCT06241456	phase 1; recruiting	pre-infusion conditioning with fludarabine/cyclophosphamide or bendamustine; additional anticancer therapy varies by cohort	https://clinicaltrials.gov/study/NCT06241456

Allogeneic products are designed as standardized, off-the-shelf biologics supported by master-cell-bank-level characterization. This structure gives them a clear scalability advantage. Donor selection, genome engineering, process qualification, release testing, and deep safety profiling can be performed once and then applied across many recipients.^54^ As a result, allogeneic platforms are particularly attractive for indications that require rapid treatment, large cell numbers, consistent dosing, or broad distribution. This advantage is especially important in time-sensitive settings. Early patient-specific iPSC manufacturing could take about a year, making autologous production difficult to align with treatment windows of days to weeks. In such settings, including some immune-cell oncology applications, off-the-shelf allogeneic supply is often more practical.^19^^,^^53^^,^^54^ However, the same donor-recipient separation that enables scale also creates the central limitation of allogeneic therapy: immune mismatch. Allogeneic products must, therefore, address alloimmunity through HLA matching, HIP engineering, pharmacologic immunosuppression, or some combination of these approaches.^19^^,^^20^^,^^54^^,^^119^ Yet, each approach has caveats. HLA matching remains population dependent, and most programs continue to require at least short-course immunosuppression. HIP engineering reduces but does not eliminate immune recognition, although early human findings are encouraging,^65^^,^^119^ and long-term persistence, efficacy, and safety across HIP-iPSC-derived products remain uncertain; moreover, the same mechanisms that shield these cells from rejection may also impair immune clearance of cells that become virally infected or malignantly transformed, a safety risk that grows with graft persistence and has motivated complementary strategies such as inducible suicide switches.^120^^,^^121^^,^^122^ Finally, because the off-the-shelf model requires extensive expansion and sustained use of a small number of cell lines to manufacture doses for many patients, those cell lines are particularly susceptible to the accumulation of genetic and epigenetic alterations during prolonged passaging.^123^^,^^124^^,^^125^^,^^126^ The number of clinically usable passages is, therefore, finite and must be bounded by genomic stability monitoring and defined release criteria.

Autologous products offer a different set of advantages and challenges. Because they are generated from a patient’s own cells, they offer intrinsic HLA compatibility and are especially compelling when durable engraftment in immunologically exposed tissues is required, compatible donor lines are unavailable, or individualized genetic correction is central to the therapeutic concept. However, this immunological advantage comes with substantial manufacturing and safety testing burdens. The costs and timeline arising from patient-by-patient reprogramming, differentiation, genomic characterization, and release testing remain key challenges.^54^ Gene-corrected autologous products may also introduce new immune targets if the restored protein is unfamiliar to the patient’s immune system, and residual genome-editing components can add further immunogenic risk.^127^^,^^128^ Emerging technologies, including chemical reprogramming, closed-cassette automation, AI-guided process control, and omics-based quality control platforms, may help reduce these barriers, but they have not yet eliminated the complexity of individualized manufacturing.

These manufacturing and immunological considerations help explain why the optimal therapeutic route increasingly depends on the clinical context. In practice, the choice between autologous and allogeneic therapies is shaped by factors such as the number of cells required, the urgency of treatment, the transplantation site, and the local immune environment. Cardiac repair may require large cell doses engrafted under peripheral immune surveillance, favoring scalable allogeneic manufacturing,^24^^,^^114^ whereas retinal therapies use far smaller grafts in the partially immune-privileged subretinal space, where autologous or HLA-matched products remain practical.^17^^,^^129^ Parkinson’s disease currently provides one of the clearest clinical contexts in which both translational routes are simultaneously active, as autologous and allogeneic iPSC-derived dopaminergic neuron programs are each in clinical testing (Tables 1 and 2) but differ in terms of the manufacturing model, immune-management strategy, and scalability. Ophthalmology illustrates the field’s historical transition from autologous iPSC-RPE transplantation toward banked and HLA-matched allogeneic RPE approaches that combine scalable manufacturing with the partial immune privilege of the subretinal space.^17^^,^^19^^,^^53^ Diabetes remains an area in which multiple translational strategies are being actively pursued, including allogeneic islet programs, emerging HIP-engineered approaches, and early autologous hCiPSC-derived therapies. Although the latter have demonstrated clinical feasibility, immunosuppression-free engraftment has yet to be established.^65^^,^^107^ In contrast, cardiac repair and immune-cell oncology currently favor allogeneic approaches because they require scalable manufacturing, standardized product release, and, for oncology applications, rapid product availability.^54^

## Conclusion and outlook

Over the past two decades, iPSC-based therapy has evolved from a conceptual breakthrough and patient-specific vision into a diverse therapeutic landscape encompassing both autologous and allogeneic approaches. As the field matured, advances in reprogramming, differentiation, manufacturing, and immune engineering gave rise to two complementary translational routes, each offering distinct advantages and limitations. Allogeneic approaches provide scalability, standardization, and off-the-shelf availability, making them particularly attractive for applications that require broad access, consistent manufacturing, or rapid treatment. Autologous approaches, in contrast, retain unique value when immune compatibility, durable engraftment, or patient-specific genetic correction is central to therapeutic success.

The future of iPSC-based medicine depends not on a single technological breakthrough but on continued progress across both pathways and on matching each approach to the clinical context in which it can provide the greatest benefit. Continued advances in chemical reprogramming, immune engineering, manufacturing automation, and quality control technologies may further improve the feasibility of both autologous and allogeneic therapies. Progress in three-dimensional tissue engineering and vascularization strategies may also expand the field beyond cell suspensions and tissue sheets toward more complex and integrated tissue constructs, although the clinical safety and efficacy of such constructs remains to be established across therapeutic areas.^54^ Ultimately, the long-term success of iPSC-based cell therapy depends on more than proving biological efficacy; it requires a careful balance of safety, manufacturability, cost, scalability, and accessibility, together with the ability to meet specific biological and clinical demands of each therapeutic setting.

## Acknowledgments

The authors thank Jinlin Wang, Chengyan Wang, Xiaojie Yu, Jingyang Guan, Xu Zhang, Jun Xu, Zilan Li, Nicole Pek, Colin Johnson, Yanglu Wang, Lin Cheng, Guan Wang, Bei Liu, Laura Monaco, Soon Yi Liew, Alonso Tapia Ramos, and Amman Klair for helpful discussions, suggestions, and editorial input during the preparation of this review.

## Declaration of interests

H.D. serves as a scientific advisor to BeiCell Therapeutics and Hangzhou Reprogenix Bioscience, Inc.

## Declaration of generative AI and AI-assisted technologies in the writing process

During the preparation of this work, the authors used Google Gemini for figure schematic generation and ChatGPT and Claude for language polishing and editing. After using these tools, the authors reviewed and edited the content as needed and take full responsibility for the content of the publication.
